# A near-complete genome assembly of cucumber line 6457 and identification of candidate gene controlling pedicel length

**DOI:** 10.1093/hr/uhaf222

**Published:** 2025-10-25

**Authors:** Yang Xie, Chenhao Zhang, Jiaojiao Zhang, Jianyu Zhao, Xiaofei Song, Xiaoxiao Lei, Lijin Fan, Xiaoli Li, Jianhua Jia, Chen Wang, Xiaolan Zhang, Liying Yan, Xiaoming Song

**Affiliations:** Hebei Key Laboratory of Horticultural Germplasm Excavation and Innovative Utilization, Hebei Higher Institute Application Technology Research and Development Center of Horticultural Plant Biological Breeding, College of Horticulture Science and Technology, Hebei Normal University of Science and Technology, Qinhuangdao 066004, China; School of Life Sciences/School of Basic Medical Sciences, North China University of Science and Technology, Tangshan, Hebei 063210, China; Hebei Key Laboratory of Horticultural Germplasm Excavation and Innovative Utilization, Hebei Higher Institute Application Technology Research and Development Center of Horticultural Plant Biological Breeding, College of Horticulture Science and Technology, Hebei Normal University of Science and Technology, Qinhuangdao 066004, China; State Key Laboratories of Agrobiotechnology, Joint International Research Laboratory of Crop Molecular Breeding, Beijing Key Laboratory of Growth and Developmental Regulation for Protected Vegetable Crops, Department of Vegetable Sciences, China Agricultural University, Beijing 100193, China; Hebei Key Laboratory of Horticultural Germplasm Excavation and Innovative Utilization, Hebei Higher Institute Application Technology Research and Development Center of Horticultural Plant Biological Breeding, College of Horticulture Science and Technology, Hebei Normal University of Science and Technology, Qinhuangdao 066004, China; Hebei Key Laboratory of Horticultural Germplasm Excavation and Innovative Utilization, Hebei Higher Institute Application Technology Research and Development Center of Horticultural Plant Biological Breeding, College of Horticulture Science and Technology, Hebei Normal University of Science and Technology, Qinhuangdao 066004, China; Hebei Key Laboratory of Horticultural Germplasm Excavation and Innovative Utilization, Hebei Higher Institute Application Technology Research and Development Center of Horticultural Plant Biological Breeding, College of Horticulture Science and Technology, Hebei Normal University of Science and Technology, Qinhuangdao 066004, China; Hebei Key Laboratory of Horticultural Germplasm Excavation and Innovative Utilization, Hebei Higher Institute Application Technology Research and Development Center of Horticultural Plant Biological Breeding, College of Horticulture Science and Technology, Hebei Normal University of Science and Technology, Qinhuangdao 066004, China; Hebei Key Laboratory of Horticultural Germplasm Excavation and Innovative Utilization, Hebei Higher Institute Application Technology Research and Development Center of Horticultural Plant Biological Breeding, College of Horticulture Science and Technology, Hebei Normal University of Science and Technology, Qinhuangdao 066004, China; Hebei Key Laboratory of Horticultural Germplasm Excavation and Innovative Utilization, Hebei Higher Institute Application Technology Research and Development Center of Horticultural Plant Biological Breeding, College of Horticulture Science and Technology, Hebei Normal University of Science and Technology, Qinhuangdao 066004, China; State Key Laboratories of Agrobiotechnology, Joint International Research Laboratory of Crop Molecular Breeding, Beijing Key Laboratory of Growth and Developmental Regulation for Protected Vegetable Crops, Department of Vegetable Sciences, China Agricultural University, Beijing 100193, China; Hebei Key Laboratory of Horticultural Germplasm Excavation and Innovative Utilization, Hebei Higher Institute Application Technology Research and Development Center of Horticultural Plant Biological Breeding, College of Horticulture Science and Technology, Hebei Normal University of Science and Technology, Qinhuangdao 066004, China; School of Life Sciences/School of Basic Medical Sciences, North China University of Science and Technology, Tangshan, Hebei 063210, China

Dear editor,

Cucumber (*Cucumis sativus* L.) is an important vegetable crop that belongs to the Cucurbitaceae family. Cucumbers are classified into four major ecological types: the Eurasian group, East Asian group, Indian group, and Xishuangbanna group. Notably, the northern HAN cucumber, a variant of the South China type belonging to East Asian group, is distinguished by its superior palatability. In recent years, its annual cultivation area in the northern solar greenhouses has consistently exhibited expansion, reflecting its growing agricultural importance. Recent advances in genomic sequencing have led to the assembly of high-quality genomes for different varieties of Cucumber. These advancements have facilitated a better understanding of the genetic basis of morphological diversity in Cucumber.

The first genome sequencing of Cucumber (‘Chinese Long’ inbred line 9930) released in 2009 was a significant milestone in understanding the genetic makeup of this important vegetable [1]. This research provided insights into the dynamics of Cucurbitaceae genome evolution and served as an important resource for Cucumber breeding. Then, the draft genome sequence of Cucumber genome of the North-European Borszczagowski cultivar (line B10) was performed [2]. Recently, the genome of ‘Chinese Long’ line was further improved, and finally achieved to the near-complete reference genome [3, 4]. Moreover, an elite Russian pickling-type inbred line was sequenced, which was also achieved to near-complete reference genome in 2025 [5]. To date, near-complete genome sequences have been assembled for the North China type (cultivar 9930) and the pickling cucumber (Gy14 v2 and CUK2021). However, genomic data for the South China type (e.g. Cucumber line 6457) remain limited, highlighting a critical gap in cucumber genetic research. Therefore, this study aims to resolve the first high-quality near-complete cucumber genome of the 6457 line, providing a higher quality genome for comparative and functional genomics research of South China type Cucumber.

To obtain the high-quality Cucumber line 6457 genome, we perform the *de novo* genome sequencing using the latest sequencing technologies, including Oxford Nanopore Technology (ONT) ultra-long reads, PacBio HiFi, Illumina and Hi-C technology. First, the genome of 6457 was estimated using K-mer analysis with 22.08 Gb of data from Illumina sequencing. The estimated genome size was 329.94 Mb, and the heterozygosity rate was 0.16%. The PacBio HiFi sequencer was adopted to generate 61.29 Gb data with coverage of 185.76×. Furthermore, Hi-C technology was employed to anchor the assembled sequences to each chromosome, and a total of 41.12 Gb (124.63×) data were obtained. The assembled genome size was 336.58 Mb, and the 295.18 Mb of sequences anchored to the 7 chromosomes (Fig. 1A). The high-quality assembled genomes with the contig N50 was 41.45 Mb. Of particular importance is that ONT ultra-long sequences (32.33 Gb, 97.99 X) were used to achieve a telomere-to-telomere (T2T) level of genome assembly. All 7 chromosomes are gap-free with 7 centromeres and 11 telomeres were detected (Fig. 1B). The read coverage rate exceeds 99.82% (Fig. 1C). The genome completeness is assessed by Benchmarking Universal Single-Copy Orthologs (BUSCO) as 98.90%, and the genome consistency quality value is 50.46.

**Figure 1:**
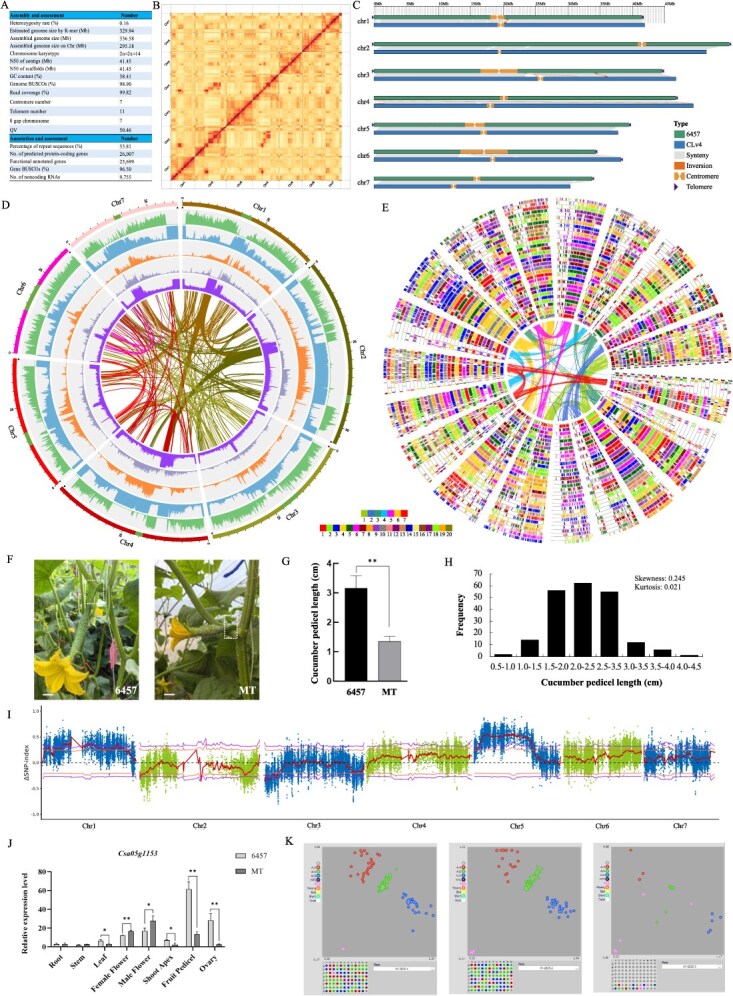
Assessment of cucumber (6457) T2T genome assembly, annotation, and evolution, and collinear analysis. **(A)** Statistics of 6457 cucumber genome sequencing, assembly and annotation. **(B)** The Hi-C contact map of 6457 genome assembly. **(C)** Chromosome map with telomere, centromere, and gap information of 6457 and CLv4 genome. **(D)** The distribution of chromosome, gene density, repeat density, Gypsy density, Copia density, and GC content, genomic synteny from the outside to the inside. **(E)** Global alignment of homologous regions. The Vvi1-19 regions of the inner circle represent chromosomes 1 to 19 of grape (*Vitis vinifera*). The layers of the outer circle represent the subgenome of the corresponding species relative to grape. **(F-G)** Pedicel length comparison between line 6457 and MT at flowering stage. The scale bar represents 1 cm. Data were analyzed by Student’s *t*-test with significance levels indicated as follows: ^*^P < 0.05, ^**^P < 0.01, ^***^P < 0.001. **(H)** Frequency distribution of pedicel length in F_2_ population. **(I)** QTL mapping for pedicel length trait in cucumber. **(J)** qRT-PCR analysis of candidate genes *Csa05g1153* associated with pedicel length. **(K)** Genotypic distribution of the *Csa05g1153*-G/A marker in parental lines and F_2_ individuals as determined by KASP assay.

Repetitive sequences accounted for 53.81% of the cucumber line 6457 genome, among which, 33.04% were belonged to DNA transposon, followed by long-terminal repeats (LTRs, 15.89%) (Fig. 1D). A total of 26 007 genes were predicted in cucumber line 6457 genome, and 96.50% of BUSCO genes (1614) were detected, indicating high completeness of gene prediction (Fig. 1D). Among all the predicted genes, over 25 699 (98.82%) genes were annotated using GO, KEGG, Pfam, Swissport, InterPro, and NR databases. Concerning, 9755 noncoding RNA was found in the cucumber line 6457 genome.

We further performed the genome collinearity analysis of cucumber (6457) and other Cucurbitaceae species. We used the ‘-icl’ program in WGDI software to evaluate the collinearity of the genome and the ‘-ci’ program to demonstrate the collinearity between species. Using grape as a reference, global alignment of homologous regions in the genomes of these species was performed (Fig. 1E). Grape has a relatively clear history of ancient polyploidization events, having undergone the whole-genome triplication event (γ) that affected most angiosperms. All 13 plant species of Cucurbitaceae underwent Whole Genome Duplication (WGD) events, so their genomes were further divided into sub-genomes. In addition, *Cucurbita argyrospora*, *Cucurbita pepo*, and *Section edule* have undergone additional whole genome duplication events, resulting in a 4:1 ratio to grape. The ratio of other Cucurbitaceae species to grape is 2:1.

Finally, we explored the candidate gene for cucumber pedicel length using this high-quality 6457 genome. Pedicel length is an important trait closely related to fruit commodity quality. To elucidate the genetic basis of this trait, the high-generation inbred lines of 6457 with long fruit pedicel (average 3.2 cm) and MT with short fruit pedicel (average 1.4 cm) were used (Fig. 1F and G). The 6457 line as female parent was crossed with MT as male parent to develop 17 F_1–1_ plants and the reciprocal cross produced 14 F_1–2_ plants. A total of 208 F_2_ plants were produced by self-crossing of F_1–1_ for an inheritance study and gene identification (Fig. 1H). Quantitative trait genetic model analysis revealed cucumber pedicel length conforms to a two-major-gene model with additive-isodominant effects (2MG-EAD; AIC = 378.483).

To identify the candidate region contributing to the cucumber pedicel length, BSA-seq was performed. Quantitative Trait Locus (QTL) analysis identified three major trait-linked intervals: chr1:1340821–13 040 685 (11.7 Mb), chr1:17821831-36 919 879 (19.1 Mb), and chr5:729112-27 503 912 (26.8 Mb) (Fig. 1I). Notably, we detected one nonsense mutations *Csa05g1153* that introduce premature termination codons in genes encoding uncharacterized proteins. Spatiotemporal expression profiling identified *Csa05g1153* displayed enriched expression in the fruit pedicel, and significantly higher expression levels in 6457 compared to that in line MT (Fig. 1J). However, no sequence variation was detected in the activation region, leaving the mechanistic basis for this differential expression unresolved. Furthermore, the *Csa05g1153* variation site exhibited co-segregation in the F_2_ population, with genotype segregation conforming to the expected 1 (A:A): 2 (A:G): 1 (G:G) Mendelian ratio, which was strongly validated (Fig. 1K).

In conclusion, we present the first high-quality near-complete genome of 6457 and identify candidate gene *Csa05g1153* potentially regulating cucumber pedicel length. This study provided us with a wealth of data resources for functional genomics studies and molecular breeding of Cucumber or even other Cucurbitaceae species.

## Data Availability

The raw BSA-seq data generated in this study have been deposited in the NCBI Sequence Read Archive (SRA) under BioProject accession PRJNA1301951 (https://dataview.ncbi.nlm.nih.gov/object/PRJNA1301951). All genome annotated data related to this study are available in the TVIR database (http://tvir2.bio2db.com).
